# Association of Prenatal Famine Exposure With Inflammatory Markers and Its Impact on Adulthood Liver Function Across Consecutive Generations

**DOI:** 10.3389/fnut.2021.758633

**Published:** 2022-01-03

**Authors:** Shiwei Yan, Jingqi Ruan, Yu Wang, Jiaxu Xu, Changhao Sun, Yucun Niu

**Affiliations:** Department of Nutrition and Food Hygiene, College of Public Health, Harbin Medical University, Harbin, China

**Keywords:** prenatal, famine exposure, transgenerational, inflammatory markers, liver function

## Abstract

Although there has been increasing recognition that famine exposure in the fetal stage damages liver function in adulthood, this deteriorated effect could be extended to the next generation remains vague. This study aimed to explore whether famine exposure was associated with liver function in the two consecutive generations, and its association with the mediation role of inflammatory markers. We analyzed the data of 2,681 participants from Suihua rural area, Heilongjiang Province, China. According to the date of birth, the participants were classified as fetal exposed and nonexposed. The F2 subjects were classified as having no parents exposed to famine, maternal famine exposure, paternal famine exposure, or parental famine exposure. In the mixed-effect models, prenatal exposure to famine was associated with the elevation of Δ aspartate aminotransferase (ΔAST) (β: 0.22, 95% *CI*: 0.01, 0.43) and Δ alanine aminotransferase (ΔALT) (β: 0.42, 95% *CI*: 0.19, 0.66) levels in F1 adults. The mediation analysis showed that the inflammatory markers including serum C-reactive protein (CRP) and tumor necrosis factor-alpha (TNF-α) might mediate the famine-liver function association. This longitudinal data were consistent with the hypothesis that the inflammatory markers explained part of the influence of prenatal famine exposure on liver function injury, and the natal mechanism was needed to be elucidated in the future study.

## Introduction

At present, chronic liver disease (CLD) has emerged as a major cause of death and illness worldwide, with millions of people dying from the liver disease each year (1–3). Until 2020, it is estimated that the number of patients with CLD, including alcoholic liver disease, non-alcoholic fatty liver disease (NAFLD), hepatitis B virus, hepatitis c virus, and end-stage liver diseases (e.g., liver cirrhosis or liver cancer), would rise to approximately 400 million in China alone, which apparently had a great impact on the global burden of liver disease. However, the high prevalence of CLD might not be simply attributed to population growth, aging, and diet and lifestyle changes in the past decades.

Since the “Developmental Origins of Health and Disease” (DOHaD) hypothesis was proposed, the topic on the effects of nutritional deprivation in the early development, especially in a sensitive period of gestation, on lifelong healthy conditions has attracted increasing attention. Extensive epidemiological studies indicated that prenatal famine exposure might increase susceptibility to obesity (4), type 2 diabetes (5), schizophrenia (6), and depression (7), all of which were associated with CLD (8–10). Recently, research into animals manifested that malnutrition in gestation had a great likelihood of influencing hepatic development (11). Unfortunately, due to the lack of available experimental data, the transgenerational effects on liver function in adult offspring were rarely reported. The Chinese famine of 1959–1961, one of the most severe catastrophes in human history, directly led to tens of millions of excess deaths nationwide (12), which offered a unique opportunity to thoroughly examine the underlying transgenerational effects of famine exposure in the fetal stage on adult liver function.

The development of liver injury was a multifactorial process, in which inflammation plays a key role. Tumor necrosis factor-alpha (TNF-α), a pleiotropic inflammatory cytokine executing biological functions, might induce the underlying mechanism to initiate apoptosis in hepatocytes further to result in liver damage. In addition, one prospective study, based on the Linxian Nutrition Intervention Trials cohort, recently showed that the C-reactive protein (CRP) concentrations positively increased the risk of both liver cancer incidence and CLD mortality (13). Although the influence of poor nutrition during gestation on inflammation, to date, has not been widely reported, the topics surrounding the association have been constantly discussed. In this study, therefore, inflammatory markers, including CRP and TNF-α, were considered as the underlying risk factors of famine-liver association.

Recent famine studies have documented the significant associations between the Chinese famine exposure in the fetal stage and increased adult risk of fatty liver (14–17), yet no study directly examined whether the underlying gestational effect on liver function could be transmitted across generations. In the current study, we, therefore, conducted a large population-based investigation from Northern China to evaluate the relation of fetal exposure to Chinese famine on adulthood liver function, and further examined the mediation effect of the inflammatory markers on the association between famine and liver function in two consecutive generations.

## Methods

### Study Population

We used longitudinal data of the participants from the Suihua Beilin rural region of Heilongjiang province, one of the worst economically developed regions in the Northeast of China, which occurred the severe famine from 1959 to 1961. In the present study, the participants at baseline, including parental generation (F1) and offspring generation (F2), were recruited on the basis of the family unit. F1 subjects born between October 1, 1959, and September 30, 1961, were defined as the fetal exposure group; F1 subjects born between October 1, 1956, and September 30, 1958, and between October 1, 1962, and September 30, 1964, were combined into the age-balanced non-exposed group. Due to the lack of the exact dates of the start and end of the Chinese famine, the adults born between October 1, 1958, and September 30, 1959, and between October 1, 1961, and September 30, 1962, were excluded to minimize misclassification bias. Correspondingly, F2 subjects were defined as no parent who was exposed to famine (neither), only a father who was exposed to famine (paternal), only a mother who was exposed to famine (maternal), and both parents who were exposed to famine (parental). In this study, the exclusion criteria were as follows: (i) a family history of chronic liver or kidney disease; (ii) a history of excessive consumption of pure alcohol, self-reported viral hepatitis, and use of medications associated with liver disease; (iii) missing data of the study questionnaire, anthropometric measurements, and laboratory parameters. Finally, 2681 subjects (1732 parents and 949 offspring) were included in this longitudinal famine study, with an average follow-up period of 5.0 years.

All the participants provided signed consent forms before data collection. The study protocol and all the procedures that comply with the Declaration of Helsinki were approved by the Ethical Committee of Harbin Medical University.

### Questionnaire, Anthropometric, and Laboratory Measurements

A standardized face-to-face interview by trained staff using a detailed questionnaire to collect the demographic and lifestyle information including age, sex (male, female), education (high school and high school below, over the high school), smoking (yes, no), drinking (yes, no), and working strength (low, medium, high). The type and frequency of physical activity were recorded. A food-frequency questionnaire (FFQ) was commonly used to collect dietary information, including frequency of intake and average consumption of each food item. The reproducibility and validity of the FFQ were assessed in a previous study (18). Besides, Food Nutrition Calculator (V1.60; Chinese Center for Disease Control) was used to estimate every participant's daily energy intake.

Anthropometric measurements, including body height, weight, and blood pressure, were obtained by experienced nurses. Weight and height were, respectively, measured to the nearest 0.1 kg and 0.1 cm with subjects wearing light clothes and no shoes. Body mass index (BMI) was calculated as weight (kg) divided by the square of the height in meters (m^2^). Referring to the guideline for prevention and control of obesity in Chinese adults, obesity was defined as BMI ≥ 28 kg/m^2^. Blood pressure was consecutively tested three times with a 1-min interval at the right arm after a 5-min rest in a sitting position using a mercury sphygmomanometer, and the mean values were used for latter data analysis.

The venous blood samples were collected after fasting for at least 10 h. After immediate centrifugation, the serum was shipped at −20°C in dry ice to the Laboratory of the Second Affiliated Hospital of Harbin Medical University. The determination of various liver enzymes activities in serum, as alanine aminotransferase (ALT), aspartate aminotransferase (AST), gamma-glutamyl transferase (GGT), alkaline phosphatase (ALP), and albumin (ALB), are used to assess the functional condition of the liver and to detect liver injury, in which ALT and AST are the most commonly used indices for evaluation of liver function (19). ΔALT was defined as ALT at follow-up–ALT at baseline, and ΔAST was defined as AST at follow-up–AST at baseline. In addition, the liver function indices and fasting plasma glucose (FPG) and 2-h postprandial glucose (2-hPG) were assessed using an automatic biochemistry analyzer (7,100; Hitachi, Tokyo, Japan). CRP was measured *via* the immunoturbidimetric method (Denka Seiken, Tokyo, Japan), and TNF-α was measured using an enzyme-linked immunosorbent assay (R&D Systems, Minneapolis, MN, USA) according to the instructions of the manufacturer.

### Definition of NAFLD

Hepatic Steatosis Index (HSI) as a surrogating index of NAFLD was considered to predict the NAFLD cases in the current study. In contrast to ultrasonography and CT, the HSI consisted of the combination of hepatic enzyme measurements, including ALT and AST, do not depend on using the imaging modalities to screen asymptomatic individuals, and conduct a simply non-invasive test to identify the patients at high risk of NAFLD with reasonable accuracy (20, 21). Moreover, the predictive accuracy has been validated in the Korean population (22).

The presence of diabetes mellitus (DM) was defined as FPG ≥ 7.0 mmol/L or 2-hPG ≥ 11.1 mmol/L or current therapy for definite diagnosis of DM. HSI ≥ 36 was considered to indicate NAFLD and derived using the following equation: HSI = 8 × ALT/AST ratio + BMI (+ 2, if DM; + 2, if female).

### Statistical Analysis

All the statistical analyses were conducted using the R 3.4.4 software (The R Foundation for Statistical Computing, Austria), and a two-tailed *P* < 0.05 was considered significant.

The continuous and categorical variables were described as the mean, mean ± SD, and a percentage (%), respectively. Considering that the subjects were enrolled by a family unit, therefore, the characteristics of the study subjects by famine exposure groups were compared using the generalized linear mixed models and χ^2^ test for continuous variables and categorical variables, respectively. As serum inflammatory markers and liver function indices exhibited the skewed disturb, natural ln transformations of them were used. The associations between the famine exposure and inflammatory markers and the variation of liver function indices were assessed using a generalized linear mixed model in both generations. The risk of NAFLD in exposure to famine was examined with the use of a generalized linear mixed model. In these models, famine exposure was the fixed factor, and the family number was the random factor. Additionally, the F2 subjects were divided into four groups according to parental famine exposure status. To analyze the relation of the exposure of fathers, mothers, and both parents with offspring liver function indices, dummy variables were established: maternal exposure, paternal exposure, and parental exposure. Model 1 was adjusted for age, sex, education, energy intake, physical activity, smoking, drinking, and working strength. Model 2 was further adjusted for BMI, SBP, and FPG.

The mediation models were constructed to investigate the role of inflammatory markers as potential mediators of the association between famine and longitudinal variation of liver function indices, after adjustment for the above covariables. Prenatal famine exposure was the predictor variable (X); inflammatory markers were the mediators (M); variation of liver function indices at baseline and follow-up was the outcome variable (Y). In total, there are four steps in mediation analysis: (1) investigating the relation of X with Y: Model Y = β_*Tot*_X; (2) investigating the relation of X with M: Model M = β_1_X; (3) investigating the relation between M and Y with X: Model Y = β_2_M + β_dir_X; (4) calculating the percentage of mediation: mediated relation (%) = (β_1_ × β_2_/β_Tot_) ×100%. (β_Tot_ = total relation, β_1_ = indirect relation, β_2_ = indirect relation, and β*dir* = direct relation).

## Results

### Population Characteristics

The characteristics of the 1,732 parents and 949 F2 offspring at baseline and follow-up are manifested in Table 1. In F1, the subjects exposed to famine in the fetal stage accounted for 47.9%. The exposed group at baseline and follow-up had a higher BMI, SBP, DBP, FPG, 2-hPG, CRP, TNF-α, AST, ALT, GGT, and ALB levels than the non-famine-exposed group. In F2, paternal exposure, maternal exposure, and both parental exposure, respectively, accounted for 21.2, 19.2, and 25.4%, and the rest of the subjects had no parental exposure. At baseline, the parental exposed group had strikingly higher BMI, SBP, FPG, 2-hPG, TNF-α, and ALT levels than the nonexposed group.

**Table 1:** Characteristics of the study variables at baseline and follow-up in parents and their offspring.

	**F1 generation**			**F2 generation**				
	**Nonexposed**	**Fetal exposed**	***P*-value**	**Neither**	**Paternal**	**Maternal**	**Both**	***P*-value**
*N*	903	829		325	201	182	241	
**Baseline**
Age (years)	52.6	53.3	0.015	26.3	26.5	26.2	26.7	0.69
Male (%)	623 (69.0)	529 (63.8)	0.022	188 (57.8)	112 (55.7)	109 (59.9)	142 (58.9)	0.85
Over high school level (%)	130 (14.4)	122 (14.7)	0.38	36 (11.1)	22 (10.9)	24 (13.2)	25 (10.4)	0.57
Low working strength (%)	251 (27.8)	209 (25.2)	0.14	88 (27.1)	50 (24.9)	44 (24.2)	61 (25.3)	0.35
Physical activity (%)	256 (28.3)	217 (26.2)	0.31	81 (24.9)	56 (27.9)	52 (28.6)	58 (24.1)	0.65
Smoker (%)	326 (36.1)	285 (34.4)	0.45	124 (38.2)	74 (36.8)	59 (32.4)	80 (33.2)	0.48
Drinker (%)	341 (37.8)	288 (34.7)	0.19	97 (29.8)	66 (32.8)	64 (35.2)	82 (34.0)	0.60
Obesity (%)	134 (14.8)	148 (17.9)	0.09	42 (12.9)	33 (16.4)	32 (17.6)	45 (18.7)	0.27
Energy intake (kcal/d)	2429 ± 1033	2492 ± 989	0.21	2571 ± 858	2465 ± 833	2556 ± 919	2499 ± 936	0.53
BMI (kg/m^2^)	24.5 ± 3.2	25.0 ± 3.2	0.007	24.1 ± 4.0	25.2 ± 3.3	25.4 ± 3.3	25.6 ± 3.1	<0.01
SBP (mmHg)	137.2 ± 22.7	141.8 ± 20.4	<0.01	124.5 ± 15.8	128.8 ± 14.3	128.2 ± 13.8	129.2 ± 14.4	<0.01
DBP (mmHg)	80.5 ± 12.8	83.0 ± 15.7	0.002	76.9 ± 11.4	77.8 ± 10.4	77.4 ± 10.4	78.0 ± 11.7	0.68
FPG (mmol/L)	4.6 ± 1.1	4.8 ± 1.2	<0.01	4.3 ± 0.5	4.4 ± 0.7	4.5 ± 0.6	4.5 ± 0.6	0.034
2-hPG (mmol/L)	6.0 ± 1.9	6.4 ± 2.6	<0.01	5.1 ± 1.3	5.4 ± 1.2	5.4 ± 1.3	5.5 ± 1.4	<0.01
CRP (mg/L)	1.8 ± 1.3	2.1 ± 1.6	<0.001	1.5 ± 1.1	1.7 ± 1.3	1.7 ± 1.3	1.8 ± 1.3	0.62
TNF-α (ng/L)	4.3 ± 2.1	5.0 ± 2.1	<0.001	3.6 ± 1.8	3.8 ± 1.9	4.0 ± 2.0	4.1 ± 2.1	0.04
AST (U/L)	24.1 ± 10.0	25.5 ± 12.4	0.028	21.1 ± 10.1	22.3 ± 10.7	22.3 ± 11.0	22.6 ± 9.7	0.083
ALT (U/L)	22.6 ± 12.3	24.4 ± 13.7	<0.01	20.0 ± 12.1	21.3 ± 11.0	21.4 ± 11.1	21.6 ± 13.1	<0.01
GGT (U/L)	24.8 ± 22.5	26.0 ± 23.9	0.15	21.6 ± 19.7	22.4 ± 21.1	22.6 ± 20.2	22.8 ± 20.2	0.67
ALP (U/L)	79.7 ± 20.1	81.5 ± 22.3	0.22	71.0 ± 18.8	72.4 ± 20.2	72.1 ± 19.7	72.7 ± 19.4	0.76
ALB (g/L)	45.0 ± 2.7	44.7 ± 2.6	0.17	46.2 ± 3.3	46.1 ± 2.9	46.0 ± 3.1	46.0 ± 2.8	0.92
**Follow-up**								
AST (U/L)	25.3 ± 10.7	27.1 ± 12.0	<0.001	22.5 ± 9.5	23.8 ± 12.1	24.1 ± 12.3	24.2 ± 11.7	0.49
ALT (U/L)	23.6 ± 13.6	25.9 ± 15.1	<0.001	20.9 ± 11.8	22.4 ± 11.9	22.7 ± 12.5	22.9 ± 12.6	<0.01
GGT (U/L)	25.7 ± 23.1	27.1 ± 24.0	0.024	22.5 ± 20.1	23.5 ± 20.2	23.8 ± 21.4	24.0 ± 20.7	0.52
ALP (U/L)	81.2 ± 20.7	83.2 ± 23.1	0.12	73.2 ± 19.8	74.9 ± 21.5	74.6 ± 20.5	75.2 ± 19.6	0.57
ALB (g/L)	44.6 ± 2.6	44.2 ± 2.6	0.002	45.5 ± 2.9	45.3 ± 2.7	45.2 ± 3.2	45.2 ± 2.7	0.52

*BMI, body mass index; FPG, fasting plasma glucose; 2-hPG, 2-h postprandial glucose; SBP, systolic blood pressure; DBP, diastolic blood pressure; TNF-α, tumor necrosis factor alpha; CRP, C-reactive protein; AST, aspartate aminotransferase; ALT, alanine aminotransferase; GGT, gamma-glutamyl transferase; ALP, alkaline phosphatase; ALB, albumin. Data are presented as mean, mean ± SD, or percentage (%). The generalized linear mixed models and χ^2^ test were used to probe for the differences in continuous variables with skewed distribution and normal distribution, and categorical variable*.

### Effects of Famine Exposure on the Level of Inflammatory Markers in Two Consecutive Generations

Table 2 presents associations of famine with CRP, and TNF-α in the two consecutive generations. As expected, in the F1 generation, the famine-exposed group had a β: 0.151 (0.099, 0.202) higher ln-TNF-α, a β: 0.100 (0.011, 0.190) higher ln-CRP, a β: 0.094 (0.019, 0.168) higher ln-TNF-α, a β: 0.193 (0.079, 0.308) higher ln-CRP in men and women, after adjustment for age, education level, smoking, physical activity, working strength, energy intake, BMI, SBP, and FPG. Nevertheless, the significant association between both parental exposure to famine and a β: 0.123 (0.026, 0.220) higher ln-TNF-α was observed only in the F2 men, after adjustment for age, education, working strength, physical activity, smoking, drinking, energy intake, BMI, SBP, and FPG.

**Table 2:** Association of famine exposure with the inflammatory markers in both generations.

	**F1 generation**		**F2 generation**					
	**Prenatal**		**Paternal**		**Maternal**		**Parental**	
	**β (95% CI)**	***P*-value**	**β (95% CI)**	***P*-value**	**β (95% CI)**	***P*-value**	**β (95% CI)**	***P*-value**
**Men**								
ln-TNF-α
Model 1	0.168 (0.117, 0.219)	<0.001	0.086 (−0.016, 0.189)	0.097	0.107 (0.003, 0.211)	0.044	0.130 (0.034, 0.225)	0.008
Model 2	0.151 (0.099, 0.202)	<0.001	0.082 (−0.021, 0.184)	0.119	0.102 (−0.003, 0.207)	0.057	0.123 (0.026, 0.220)	0.013
ln-CRP								
Model 1	0.107 (0.019, 0.195)	0.017	0.056 (−0.119, 0.231)	0.533	0.037 (−0.144, 0.218)	0.687	0.074 (−0.092, 0.239)	0.383
Model 2	0.100 (0.011, 0.190)	0.028	0.054 (−0.121, 0.230)	0.545	0.039 (−0.142, 0.219)	0.676	0.062 (−0.104, 0.229)	0.461
**Women**								
ln-TNF-α
Model 1	0.098 (0.024, 0.171)	0.009	0.033 (−0.084, 0.151)	0.579	0.078 (−0.048, 0.203)	0.224	0.090 (−0.025, 0.205)	0.123
Model 2	0.094 (0.019, 0.168)	0.014	0.031 (−0.091, 0.153)	0.617	0.075 (−0.055, 0.205)	0.258	0.087 (−0.034, 0.208)	0.157
ln-CRP								
Model 1	0.205 (0.093, 0.318)	<0.001	0.101 (−0.106, 0.308)	0.337	0.092 (−0.128, 0.313)	0.410	0.130 (−0.073, 0.332)	0.209
Model 2	0.193 (0.079, 0.308)	<0.01	0.100 (−0.115, 0.315)	0.360	0.088 (−0.142, 0.317)	0.452	0.126 (−0.088, 0.339)	0.248

*TNF-α, tumor necrosis factor alpha. CRP, C-reactive protein. TNF-α and CRP were ln-transformed. In the F1, β (unstandardized coefficient) and their 95% CIs were calculated with the use of a generalized linear mixed model with prenatal famine exposure (yes or no) as the fixed factor and family number as the random factor. Model 1 was adjusted for age, education, working strength, physical activity, smoking, drinking, and energy intake. Model 2 was adjusted for Model 1 plus BMI, SBP, and FPG. In the F2, β (unstandardized coefficient) and their 95% CIs were calculated with the use of a generalized linear mixed model with parental famine exposure (paternal, maternal, or one or both parents) as the fixed factor and family number as the random factors. Model 1 was adjusted for age, education, working strength, physical activity, smoking, drinking, and energy intake. Model 2 was adjusted for Model 1 plus BMI, SBP, and FPG*.

### Effects of Famine Exposure on the Risk of NAFLD in Two Consecutive Generations

As shown in Table 3, in the F1 generation, the risk of NAFLD among the women who were prenatally exposed to famine was 1.56 (1.01, 2.43); However, no significant association of NAFLD with maternal, paternal, or parental exposure to famine was found in men, after adjustment for age, education level, smoking, physical activity, working strength, energy intake, SBP, and FPG. In the F2 generation, regardless of men and women, there was no significant association of NAFLD with maternal, paternal, or both parental exposure to famine, adjusted for age, education level, smoking, physical activity, working strength, energy intake, SBP, and FPG, due to the relatively young age in adult offspring.

**Table 3:** Odds ratios (*OR*s) (95% *CI*) for parental and offspring non-alcoholic fatty liver disease (NAFLD) among the different famine exposure groups.

	**F1 generation**		**F2 generation**				
	**Nonexposed**	**Exposed**	**Neither**	**Paternal**	**Maternal**	**Parental**	**One or both**
**Men**							
Cases/Total	186/623	175/529	40/188	21/112	19/109	30/142	70/363
Model 1	1.00 (Ref)	1.13 (0.88, 1.46)	1.00 (Ref)	0.83 (0.45, 1.53)	0.74 (0.40, 1.40)	0.96 (0.55, 1.68)	0.85 (0.54, 1.34)
Model 2	1.00 (Ref)	1.08 (0.84, 1.41)	1.00 (Ref)	0.82 (0.44, 1.51)	0.72 (0.38, 1.36)	0.92 (0.52, 1.62)	0.82 (0.52, 1.31)
**Women**							
Cases/Total	50/280	72/300	14/137	9/89	8/73	12/99	29/261
Model 1	1.00 (Ref)	1.64 (1.06, 2.53)^*^	1.00 (Ref)	0.91 (0.38, 2.21)	0.99 (0.39, 2.51)	1.19 (0.51, 2.75)	1.03 (0.52, 2.05)
Model 2	1.00 (Ref)	1.56 (1.01, 2.43)^*^	1.00 (Ref)	0.87 (0.35, 2.17)	0.95 (0.37, 2.44)	1.13 (0.48, 2.69)	0.98 (0.48, 2.00)

*In the F1 generation, OR and their 95% CIs were calculated with the use of a generalized linear mixed model with prenatal famine exposure (yes or no) as the fixed factor and family number as the random factor. Model 1 was adjusted for age, education, working strength, physical activity, smoking, drinking, and energy intake. Model 2 was adjusted for Model 1 plus SBP and FPG. In the F2 generation, OR and their 95% CIs were calculated with the use of a generalized linear mixed model with parental famine exposure (paternal, maternal, or one or both parents) as the fixed factor and family number as the random factors. Model 1 was adjusted for age, education, working strength, physical activity, smoking, drinking, and energy intake. Model 2 was adjusted for Model 1 plus SBP and FPG*.

* *P <0.05*.

### Association of Famine Exposure With ΔALT and ΔAST in Two Consecutive Generations

The results of a generalized linear mixed model that tested the association of famine exposure with ΔALT and ΔAST in the two consecutive generations is summarized in Table 4. In the F1 generation, prenatal exposure to famine was significantly associated a β: 0.42 (0.19, 0.66) higher ΔALT and a β: 0.22 (0.01, 0.43) higher ΔAST, adjusted for age, sex education, working strength, physical activity, smoking, drinking, energy intake, BMI, SBP, and FPG. Nevertheless, no remarkable association between exposure to famine and ΔALT and ΔAST was observed in adult offspring, after adjustment for age, sex education, working strength, physical activity, smoking, drinking, energy intake, BMI, SBP, and FPG.

**Table 4:** Associations with famine exposure and parental and offspring Δ alanine aminotransferase (ΔALT) and Δ aspartate aminotransferase (ΔAST) in adulthood.

	**F1 generation**		**F2 generation**	
	**Coefficient (95% CI)**	***P*-value**	**Coefficient (95% CI)**	***P*-value**
ΔALT				
Model 1	0.49 (0.26, 0.73)	<0.001	0.31 (0.01, 0.61)	0.041
Model 2	0.42 (0.19, 0.66)	<0.01	0.27 (−0.03, 0.57)	0.082
ΔAST				
Model 1	0.27 (0.06, 0.48)	0.013	0.20 (−0.08, 0.47)	0.16
Model 2	0.22 (0.01, 0.43)	0.045	0.18 (−0.10, 0.46)	0.21

*In the F1, the coefficients and their 95% CIs were calculated with the use of a generalized linear mixed model with prenatal famine exposure (yes or no) as the fixed factor and family number as the random factor. Model 1 was adjusted for age, sex, education, working strength, physical activity, smoking, drinking, and energy intake. Model 2 was adjusted for Model 1 plus BMI, SBP, and FPG. In the F2, coefficients and their 95% CIs were calculated with the use of a generalized linear mixed model with parental famine exposure (neither or one or both parents) as the fixed factor and family number as the random factor. Model 1 was adjusted for age, sex, education, working strength, physical activity, smoking, drinking, and energy intake. Model 2 was adjusted for Model 1 plus BMI, SBP, and FPG*.

### Mediation Analysis

**Figure 1:**
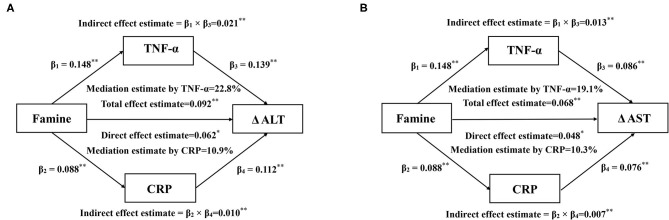
Mediation effects of serum CRP and TNF-α on the association between prenatal famine and Δ ALT **(A)** and Δ AST **(B)**. Data are standardized regression coefficients with adjustment for age, sex, education, working strength, physical activity, smoking, drinking, energy intake, BMI, SBP, and FPG. *P < 0.05, **P < 0.01 for coefficients being different from 0.

We found a significant association of prenatal exposure to famine with ΔALT and ΔAST, therefore, establishing the mediation model further to probe the underlying association. The mediation effect of serum CRP and TNF-α on the association between famine exposure in the fetal stage and ΔALT and ΔAST is shown in Figure 1. The total effect of famine on ΔALT (Figure 1A) and ΔAST (Figure 1B) measured as standardized regression coefficient (β_Tot_ = 0.092, *P* < 0.001) and (β_Tot_ = 0.068, *P* = 0.005) was, respectively, estimated in the model with adjustment for age, sex, education, working strength, physical activity, smoking, drinking, energy intake, BMI, SBP, and FPG. The β_1_ to β_4_ were used to calculate the indirect effect of TNF-α (β_ind_ = 0.021, *P* < 0.001 for ΔALT; β_ind_ = 0.013, *P* = 0.002 for ΔAST) and CRP (β_ind_ = 0.010, *P* = 0.004 for ΔALT; β_ind_ = 0.007, *P* = 0.017 for ΔAST). The percentages of the total effect on ΔALT and ΔAST mediated by TNF-α and CRP were estimated at 22.8, 19.1, 10.9, and 10.3%, respectively.

## Discussion

In this large longitudinal cohort of 2,681 participants from Northern China, there was a significant association between prenatal famine exposure and the risk of NAFLD only in adult women. Additionally, we observed that the inflammatory markers, including CRP and TNF-α, considerably mediated the influence of prenatal exposure to famine on ΔALT and ΔAST in the mediated-analysis model.

Intriguingly, it has become apparent that the consequences of early-life malnutrition on nutrient metabolisms, such as insulin resistance and fat dysfunction which are closely related to NAFLD, differ in men and women (23–25). The findings of the current study were in agreement with the two previous studies (14, 15), which showed that the impact of prenatal and postnatal famine exposure on the risk of NAFLD was more pronounced in women rather than in men. In addition, there were animal models revealing the impact of gestational nutritional conditions on the liver (26, 27). It is noteworthy that the mortality selection hypothesis is viewed as a reasonable explanation for the difference of famine exposure on the sex-specific effect. In a severe famine period, the male death rate in infancy often exceeds that for female, which masks the true health impact of famine on men (28). In other words, men subjects might be a healthier portion of the total cohort exposed to famine. Additionally, in the influence of Chinese traditional notion, sons generally achieved more food availability and welfare than daughters, especially in economically underdeveloped regions (29). Famine-exposed women in infancy and childhood, therefore, might keep in long-term malnutrition, which contributes to worsening the health conditions in later life.

While prenatal factors, such as low birth weight and premature birth, were risks for causing metabolic disorders, postnatal growth always plays a prominent role in the metabolic disease process. There is robust evidence that rapid weight gain during the postnatal period might increase later metabolic disease risks, with numerous observational studies showing that faster early growth is strongly associated with obesity, insulin resistance, NAFLD, and other metabolic syndrome risk factors (30). Because growth acceleration to recovery from postnatal starvation was often accompanied by the events of severe childhood malnutrition, this might provide an explanation for the increased risk of metabolic disease among those exposed to postnatal malnutrition.

A number of epidemiological investigations have highlighted the role of nutritional deprivation in gestation on liver health. This association may be explained by the “fetal programming theory” that permanent structural adaptations made by the fetus in response to an adverse nutritional environment during the critical stages of fetal development led to a series of metabolic diseases. Hence, when a pregnant mother was undernourished, her body fat mobilization could be facilitated to increase energy supply for fetal growth, thereby resulting in severe lipid metabolism disorder. Several animal studies supported this point and found that intrauterine malnutrition has a likelihood of eliciting oxidative stress (OS) through inducing lipid metabolism disorder to inhibit fetal hepatic development (26, 27). Mounting evidence suggested that OS and inflammation were the important pathogenetic factors in many forms of liver diseases, regardless of etiology. In the liver, OS is one of the critical pathogenic processes that mostly relates to the disorder of redox homeostasis. When the state in which intracellular redox balance tends to the oxidizing milieu, excessive reactive oxygen species (ROS) will be accumulated. The main source of ROS in liver diseases originates from Kupffer cells, one of the resident innate immune cell populations, that might constantly produce inflammatory markers, such as TNF-α, though NF-κB mediated mechanism as well as activated it to result in the production of oxidants and consequently hepatocellular damage and metabolic dysregulation (31–33). Moreover, TNF-α may involve in executing the biological functions as diverse as inflammatory responses, hepatocellular proliferation, and apoptosis, and it is the latter that is a salient characteristic feature of many forms of liver disease. As the number of surviving hepatocytes diminishes, this process can contribute to a progressive deterioration of liver function. Additionally, CRP plays a pivotal role in the progress of hepatic injury. It is reported that the CRP concentrations were positively associated with the elevation of liver enzymes (19). Intriguingly, in a most recent study, both CRP and GSH, well known as an antioxidant, have been demonstrated to exert synergistic effects in the association of severe hepatic diseases (34). Since the possible interactions between these two inflammatory factors in mediating the famine-related liver injury remain unclear, the conjoint effects of CRP and TNF-α on the severity of a liver injury cannot be ignored.

Our results pointed out that exposure to famine in the fetal stage markedly increased the levels of serum CRP and TNF-α. Thus, the inflammatory markers, in the current study, were considered as possible risk factors of the famine-liver function association. To verify this suspicion, we did a mediation analysis using the inflammatory markers as mediators to examine the effect of prenatal famine exposure on liver function. The findings of the current study showed that the association of prenatal exposure to famine and ΔALT and ΔAST was significantly mediated by CRP and TNF-α. This suggested that control of intrauterine malnutrition-driven inflammatory markers might be one of the pathways to prevent liver function injury. Some other mechanisms linking prenatal exposure to famine with liver function injury might exist and need to be estimated in further studies.

Up till now, few research directly investigated the underlying transgenerational effects of early-life malnutrition on the impact of liver function. To our knowledge, it is still sparse that a statistical link between famine exposure in the growth stage and the elevation of liver function indices might be transmitted to the next generation. Here, our population-based investigation is the first to provide direct evidence that prenatal exposure to famine could have detrimental effects on the levels of liver function in both generations. Although the precise mechanisms responsible for the transgenerational effect of fetal famine exposure on liver function are not yet fully characterized, existing plausible interpretations of these data can be accepted. Prior reporters showed that the variations of maternal nutritional status in gestation might induce the formation of adverse metabolic phenotypes in the offspring (35–37). And there were indications that the inheritance of phenotypes persisted in multiple generations *via* epigenetic modifications through the germline of parents, such as DNA methylation (DNAm). For gestational women who are exposed to famine, their daughter and grandchildren could be directly influenced by exposure to the intrauterine insult. Indeed, evidence from the animal models supported that maternal nutritional constraint during pregnancy caused the epigenetic alterations in the liver and this is transmitted to the next generation (38–40). Thus, combined with the above findings, we draw a reasonable inference that the transgenerational association between F1 prenatal famine exposure and F2 impaired liver function was likely attributed to the transgenerational alteration of epigenome pattern through the F1 germline.

There are several strengths in the current study. To our knowledge, this large population-based cohort study first explored the association of famine exposure on adult liver function across consecutive generations. Meanwhile, we for the first time evaluated the effect of inflammatory markers as mediators on famine-liver function in adulthood. Some limitations should be taken into consideration when interpreting these findings. First, the periods of famine exposure, due to lack of accurate famine date of beginning and ending, was unlikely to be as precisely estimated as in the Dutch famine study. Considering that the Chinese famine broke out nationwide, the age-balanced controls were hard to be found. Thus, to minimize the influence of age bias, the individuals born during the pre-famine and post-famine were combined as nonexposed famine in this study. Second, despite that the proportion *via* mediation estimate reported in the current study might overestimate the real effect of inflammatory markers on famine-liver function association, the statistical model offers possible methods that warrant further exploration. Third, due to the short follow-up period of the current survey, this might be the main reason for the unremarkable changes in ALT and AST. Hence, we intend to keep on the regular return visits to collect more longitudinal data in terms of liver function in the future study. Fourth, we tried to define NAFLD by HSI instead of pathology or ultrasonography, therefore, the accuracy of diagnosis of NAFLD was difficult to be assured. The last limitation is that sample data of this study were merely limited in northern China, so it may not represent the general population of China in consideration of the severity of famine and living standard discrepancy in each area. Hence our findings were required to replicate in another Chinese famine cohort.

## Conclusion

Our study noted that fetal-exposed starvation increased the risk of NAFLD only in F1 adult women. Although no prominent association was found in their offspring, certain attention ought to be paid to the burden of gestational malnutrition to the liver in considering the age growth. Additionally, the inflammatory markers mediated a considerable proportion of the association between prenatal famine exposure and ΔALT and ΔAST in adulthood. This provided a novel pathway to evaluate the effect of early-life nutritional constraints on the progression of liver injury.

## Data Availability Statement

The raw data supporting the conclusions of this article will be made available by the authors, without undue reservation.

## Ethics Statement

The studies involving human participants were reviewed and approved by Harbin Medical University. Written informed consent to participate in this study was provided by the participants' legal guardian/next of kin. Written informed consent was obtained from the individual(s), and minor(s)' legal guardian/next of kin, for the publication of any potentially identifiable images or data included in this article.

## Author Contributions

CS and YN conceived and designed the idea for the study. JR arranged a series of procedures to achieve population data. SY analyzed the data, interpreted the results, and wrote the manuscript. All authors were responsible for revising the manuscript and approving the final version.

## Funding

This research was supported by the Applied Technology Research and Development Plan of Heilongjiang Province (GA20C012 to YN).

## Conflict of Interest

The authors declare that the research was conducted in the absence of any commercial or financial relationships that could be construed as a potential conflict of interest.

## Publisher's Note

All claims expressed in this article are solely those of the authors and do not necessarily represent those of their affiliated organizations, or those of the publisher, the editors and the reviewers. Any product that may be evaluated in this article, or claim that may be made by its manufacturer, is not guaranteed or endorsed by the publisher.
